# Responses of a soil-inhabiting collembolan (*Entomobrya proxima* Folsom) to organic fertilizer addition illustrated by functional traits and gut bacterial community

**DOI:** 10.3389/fmicb.2025.1509447

**Published:** 2025-02-04

**Authors:** Xinyue Yang, Gang Li, Weiming Xiu

**Affiliations:** Agro-Environmental Protection Institute, Ministry of Agriculture and Rural Affairs, Tianjin, China

**Keywords:** *Entomobrya proxima* Folsom, functional traits, gut microbiota, organic fertilizer, concentration

## Abstract

**Introduction:**

Organic fertilizer offers significant advantages for sustainable agricultural development compared to inorganic fertilizers and is increasingly becoming the predominant fertilizer strategy. Functional traits and gut microbiota of soil fauna are recognized as potential indicators of environmental changes. However, there is a dearth of research examining the correlation between functional traits and intestinal microorganisms in response to organic fertilizer.

**Methods:**

In this study, we selected *Entomobrya proxima* Folsom, a predominant soil collembolan species found in cropland across North China, as our subject of study. We set treatments with no organic fertilizer (CK) and three different concentrations of organic fertilizer at 1% (O1), 6% (O2), and 10% (O3). Stereomicroscopy and high-throughput amplicon sequencing were employed to elucidate the response of soil fauna to organic fertilizer through host functional traits and associated gut microbial communities.

**Results:**

The results indicated that the impact of organic fertilizer on the functional traits of collembolans was closely linked to the input concentration. Specifically, low input concentrations positively influenced all functional traits of *Entomobrya proxima* Folsom; conversely, higher input concentrations exerted an overall detrimental effect. For the gut bacterial community, the addition of organic fertilizer resulted in a significant decrease in abundance, adversely affected *α*-diversity, and significantly altered the structure of the gut bacterial community compared to CK. However, there was no significant effect of fertilizer concentration on these three parameters. The composition of the gut bacterial community varied due to the addition of organic fertilizer, with significant changes observed in the relative abundances of six phyla and three genera. Furthermore, body length and foreleg length may serve as potential indicators for characterizing the proportions of *Alcanivorax* and *Sphingobacterium* of gut bacterial community. Additionally, the assembly process of the gut bacterial community was strongly influenced by the amount of organic fertilizer added; this led to a narrowing niche width that is believed to contribute to an increase in species richness.

**Discussion:**

In conclusion, adding organic fertilizer exerted multiple impacts on soil fauna, with effect sizes related to its concentration. These findings provide insights for conserving soil animals while maximizing their ecological functions and offer perspectives on optimizing sustainable agricultural management practices.

## 1 Introduction

The application of inorganic fertilizer represents a typical approach of highly intensive agricultural management (Wang et al., 2021), which leads to changes in soil chemical properties, structure and microbial communities, and imbalance of nutrient elements, resulting in an increasingly prominent issue of biodiversity reduction in the agroecosystems (Li et al., 2021; Wang et al., 2022). Organic fertilizer, as an essential fertilizer resource, has been proven to exert positive effects on many soil animal groups and can increase their abundance and diversity via feeding stimulation, reproduction enhancement, etc. (Geng et al., 2020; Ren et al., 2020; Niu et al., 2024). And organic manure produced by livestock and poultry manure also can stabilize soil structure, enhance nutrient cycling, promote microbial biomass (Marsland et al., 2019; Ling et al., 2016), improve food resource availability for soil fauna, and increase the efficiency of nutrient supply to plants (Lapa et al., 2011). Currently, in order to foster a more harmonious agricultural environment and enhance crop productivity with aim to enhance biodiversity and promote global food security, numerous countries worldwide are actively developing technical solutions for sustainable agricultural intensification (Kumar et al., 2023). Organic fertilizer is more beneficial to agricultural sustainable development than inorganic fertilizer, which has attracted wide attention (Li et al., 2018; Diacono and Montemurro, 2010).

The realization of soil ecosystem functions is intricately linked to the presence and activities of soil animals (Thakur and Geisen, 2019; Osler and Sommerkorn, 2007). Distinct groups of soil fauna play specific roles, which enhance nutrient availability and maintain soil system health by stimulating organic matter mineralization and decomposition, forming soil pore structures, enhancing microbial activity, and inhibiting pathogenic microbial proliferation (Coleman et al., 2024). On the one hand, soil animals serve as key drivers in various soil processes, significantly influencing agricultural production. On the other hand, soil fauna is significantly influenced by agricultural management practices (Zhu et al., 2017). Currently, related findings were mainly based on data collected from studies centering on density and biomass of soil faunal population. However, the mechanisms underlying individual soil animal responses to agricultural measures are poorly studied.

**Figure 1:**
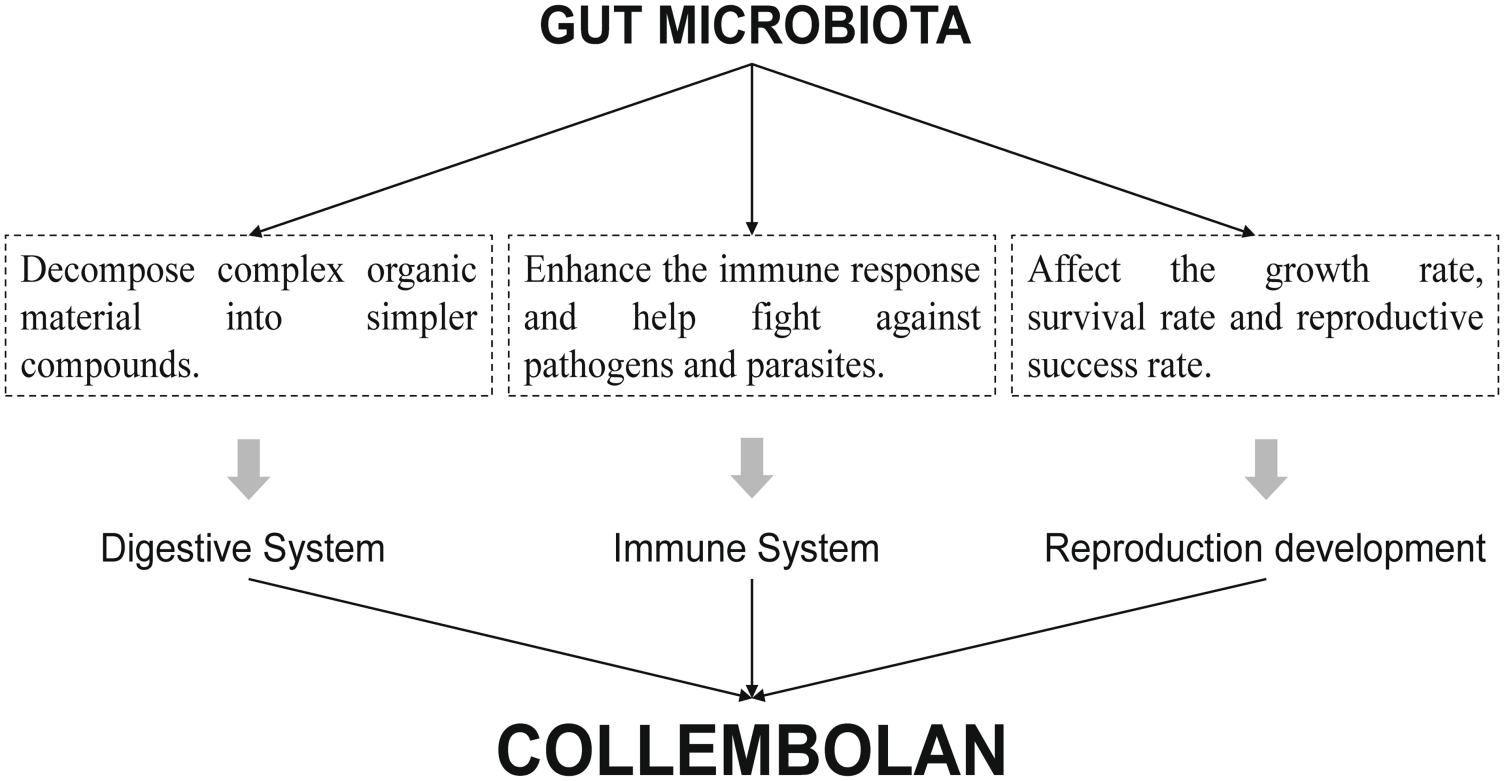
The importance of gut microbiota to collembolans.

Collembolans are a quantitatively and functionally important factor in most terrestrial ecosystems. Together with mites and some other less abundant groups, they build up the group of microarthropods (Tebbe et al., 2006). Most collembolans live in soil or leaf litter covering the soil surface (Tebbe et al., 2006) and are widely distributed worldwide (Potapov et al., 2023). As an important link in the food web, collembolans feed on algae, plant roots, soil bacteria, fungi and other microorganisms, and also decompose animal and plant residues and humus. Besides, due to prevalence in the topmost soil layers (Potapov et al., 2016) and habitat-environment-dependent life activities (Parisi et al., 2004), collembolans are extremely susceptible to soil environmental disturbances (Ding et al., 2019a). One study revisited the relevance of arthropods and their associated microorganisms in the refractory organic compound cycle, and proposed the concept of Ecosystem Holobiont (EH) and indicated that the EH is the functional unit characterized by carrying out key ecosystem processes (Schapheer et al., 2021). Therefore, the study of soil animal gut microbiota has received extensive attention. The intestinal tract of soil animals is inhabited by a large variety of microbial populations (Pass et al., 2015; Berg et al., 2016), which can contribute to resource utilization of the host (Thakuria et al., 2010), regulate the transmission of host pathogens o enhance immunity (Bahrndorff et al., 2016), and is closely related to the reproduction and development of collembolans. Besides, because of the sensitivity of the gut microbiome, whose alterations can reflect disturbances in the soil environment within their habitats (Chao et al., 2019), which is often regarded as an indicator for effective characterization of environmental changes (Ding et al., 2019b) (Figure 1). Nowadays, numerous studies have investigated the responses of collembolan gut microbial communities to soil pollutants (Chelinho et al., 2019) such as microplastics (Ju et al., 2019) and heavy metals (Zhu et al., 2018; Wang et al., 2019). However, mechanisms that target the gut microbiota of collembolans in response to agricultural practices are rare, let alone elucidations of the impact pathways and effect sizes of relevant measures.

Functional traits, as the direct reflection of ecological functions performed by collembolans in soil ecosystem, can more intuitively demonstrate inhabiting environment effects on soil collembolans (Pey et al., 2014; Sun J. et al., 2024; Sun X. et al., 2024), which have the potential to determine impacts on ecosystem processes in combination with biological fitness (Mcgill et al., 2006). For instance, antenna represents a vital sensory organ for collembolans with capabilities of location for food resources and communication among individuals, and longer antennae are more conducive to sensing environmental changes (Winck et al., 2017). Leg length stands for an indicator of exercise ability, determining the time for an individual to reach food resources (Sun J. et al., 2024; Sun X. et al., 2024). As such, exploring the effects of organic fertilizer addition on the soil collembolans at the levels of associated microbiome and functional trait will shed light on the intrinsic and extrinsic impacts of organic fertilizer on soil fauna, and help sort out linkages between effect processes, as well as unveil the underlying mechanisms.

Therefore, in the current study, *Entomobrya proxima* Folsom, a collembolan species from farmland in North China with entire high-quality genomic information (Jin et al., 2023) and numerical dominance, was selected to investigate the effects of organic fertilizer addition on soil faunal functional traits and gut bacterial community, and to conduct correlation analysis, aiming to explore the linkage response of collembolans to organic fertilizer application from phenotypic (functional traits) and physiological (gut bacterial community) aspects.

## 2 Materials and methods

### 2.1 Test collembolan

The test collembolan, *Entomobrya proxima* Folsom (Collembolan: Entomobryidae) (abbreviated as *E. proxima* below), used in this study, was isolated from farmland at Wuqing Experiment Station for Field Observation of Farmland Ecosystem, Chinese Academy of Agricultural Sciences (Tianjin), which was categorized as the topsoil species within collembolan ecological categories and has been continuously reared in the lab for 2 years after domestication. Prior to culture, species identification has been conducted using both morphological and molecular approaches to determine the phylogenetic affiliation. The feeding of *E. proxima* was carried out referring to the standardized methods of the OECD (2016). The specimens were under incubation in Petri dishes containing a mixture of plaster of Paris and activated charcoal (ratio 8:1 w/w), which were kept in an incubator at 21 ± 2°C and 75% relative humidity with a 16 h dark /8 h light photoperiod (800 lux). Yeast was provided as food for the collembolans every 6 days, and any uneaten feed was removed immediately. Additionally, ultrapure water was added to each Petri dish every 3 days to sustain ambient humidity.

### 2.2 Experimental design

Direct dietary exposure was employed to simulate collembolan feeding scenario under organic fertilization. Four treatments were established in the present study: feed without organic fertilizer (treatment CK), feed containing 1% organic fertilizer (treatment O1), feed containing 6% organic fertilizer (treatment O2), and feed containing 10% organic fertilizer (treatment O3). Each treatment was set 6 parallel petri dishes to give a total of 24 dishes. The feed used in the experiment was initially made from a uniform mixture of yeast, specific proportions of organic fertilizer as above, and ultrapure water. After frozen at −80°C, the feed underwent freeze-drying and thorough grinding, and was finally preserved at −80°C for subsequent use. The organic fertilizer tested in this study was manufactured by Lianhua Health Industry Group Co., Ltd. (Xiangcheng, Henan province, China). The organic fertilizer consists of organic matter (≥45%) and other elements (N + P_2_O_5_ + K_2_O ≥ 5%).

Prior to the exposure experiment, *E. proxima* were synchronized according to OECD guidelines (OECD, 2016) to minimize the impacts of discrepancy of age and body size on final results. At the onset, 13–15-day-old *E. proxima* (20 individuals) were transferred into each petri and fed with feed containing corresponding ratios of organic fertilizer. The culture conditions throughout the whole experimental period were set as described above, and replacement of feed and supplement of sterile water were on schedule.

### 2.3 Sample collection and functional trait measurement

After exposure for 120 days, faunal sample collection was conducted. Twenty adult collembolans were randomly selected from same treatment and fixed on the slide with acacia gum for determination of body length, body width, antennae length, fore leg length, middle leg length, and hind leg length using stereomicroscope.

### 2.4 DNA extraction of gut microbiota

When sampling, the 6 parallel petri dishes from the same treatment were mixed in pairs to form 3 replicates (Zhu et al., 2018), on the one hand to reduce errors, on the other hand to provide sufficient samples Then, 10 grown-up collembolans were randomly chosen from each replicate and transferred into a 1.5 mL centrifuge tube. The gathered collembolans were washed three times with the 2.5% sodium hypochlorite solution, and subsequently washed five times using sterilized water in order to eliminate the influence of microbiota from body surface on the analysis of intestinal bacterial community (Zhu et al., 2018). DNA isolation was performed using the DNeasy^®^ Blood & Tissue Kit (Qiagen, Germany). According to the manufacturer’s instructions, reagent ATL, protease K, AL, anhydrous ethanol, AW1, AW2 and AE were added into the kit successively. DNA was obtained through incubation and centrifugation after corresponding procedures. A total of 12 tubes of DNA were obtained from 4 treatments (three replicates for each treatment). After DNA extraction, 1.0% agarose gel electrophoresis was conducted to determine the integrity of extracted DNA, and subsequently spectrophotometric analysis was applied to check the concentration and quality of extracted DNA using a NanoDrop ND-2000 spectrophotometer (Thermo Fisher Scientific, United States). The DNA samples were divided into two parts, one for quantification of gut bacterial abundance, and another for amplicon high-throughput sequencing of the gut bacterial community. All DNA samples were stored at −80°C for further study.

### 2.5 Determination of gut bacterial community abundance

Gut bacterial community abundance was quantified using real-time fluorescent qPCR with forward primer EUB338: 5’-ACTCCTACGGGAGGCAGCAGG-3′ and reverse primer EUB518: 5’-ATTACCGCGGCTGCTGG-3′. The assays were conducted on a CFX96 Real-time PCR system (Bio-Rad, United States). The 25 μL of qPCR reaction mixture consisted of 12.5 μL of TB Green™ Premix Ex Taq™ (Tli RNaseH Plus) (TaKaRa, Japan), 0.5 μL of each primer, 0.05 μL of T4 gene 32 protein (Roche, Canada), 1.0 μL of 10-fold diluted template DNA (about 4.6–9.0 ng), and ddH_2_O. Three amplicons were performed on each tube of DNA, so a total of 36 amplicons from 4 treatments were performed. The qPCR reactions were conducted following the below procedure: an initial denaturation at 95°C for 2 min, followed by 40 cycles of denaturation at 95°C for 30 s, annealing at 55°C for 30 s, and elongation at 72°C for 30 s. Fluorescence signals were collected during the elongation phase at 72°C. The no template control (NTC) was also included in the qPCR analysis as negative controls. Standard curves were generated using serial 10-fold dilutions of plasmid, and three parallel amplifications were conducted for each dilution gradient. In this study, the amplification efficiencies were above 95% and the *R*^2^ values were above 0.99.

### 2.6 PCR amplification, high-throughput sequencing, and bioinformatic analysis

The extracted DNA samples mentioned above were sent to Majorbio Bio-Pharm Technology Co. Ltd. (Shanghai, China) for amplicon high-throughput sequencing of intestinal bacterial community. In brief, the barcoded primer set 515F/806R (forward 5’-GTGCCAGCMGCCGCGGGG-3′, reverse 5’-GGACTACHV GGGTWTCTAAT-3′) was used to amplify the hypervariable V3–V4 region of the bacterial *16S rRNA* gene (Duan et al., 2025). The PCR reaction condition was as follow: an initial denaturation at 95°C for 3 min, followed by 29 cycles comprising denaturation at 95°C for 30 s, annealing at 53°C for 30 s, and extension at 72°C for 45 s. For the PCR reaction mixture, a total volume of 20 μL was prepared consisting of Pro Taq buffer (10 μL), each primer (0.8 μL), template DNA (10 ng), and sterile ddH_2_O (Majorbio, Shanghai, China). Following amplification, the PCR products were analyzed by agarose gel electrophoresis (2%) to assess their size. The DNA bands with the required fragment size were cut and recovered. Purification was performed using a gel purification kit. The concentration of the purified DNA samples was determined. After the library was constructed by mixing according to the equal molar number, and eventually sequenced on the Illumina MiSeq platform (Illumina, San Diego, United States) through the paired-end sequencing (2 × 300) method (Majorbio, Shanghai, China).

Optimized date was obtained through splitting, quality control, and merge of the raw data. The Divisive Amplicon Denoising Algorithm 2 (DADA2) was then employed for the sequence denoising to remove low-quality and ambiguous reads. Sequences were clustered into Amplicon Sequence Variants (ASVs) at 100% sequence similarity. One representative sequence for each ASV was chosen for subsequent analysis, and taxonomic annotations of bacterial ASVs were done based on SILVA database (release 138). The following metrics were calculated as indicators of *α*-diversity of gut bacterial community by comparing the level of bacterial ASV diversity: Chao1 (community richness) and Shannon index (community diversity). Principal coordinate analysis (PCoA) based on the Bray–Curtis dissimilarity was used to evaluate *β*-diversity of gut bacterial community and PERMANOVA test was performed to test whether the samples differed significantly.

### 2.7 Statistical analysis

The significance of differences between treatments was assessed by one-way analysis of variance (one-way ANOVA) at a significance level of *p* = 0.05 with Duncan HSD test. ANOVA was performed using *EasyStat* package in R 4.2.3. Visualization of the abovementioned parameters was conducted using Origin 2021, apart from intestinal bacterial community composition that was plotted in R 4.2.3. Additionally, the PERMANOVA tests and Principal Coordinates Analysis (PCoA) based on Bray–Curtis dissimilarity were conducted in R 4.2.3 with *vegan* package. To explore relationship between functional traits and intestinal bacterial community abundance and diversity, as well as composition (main phylum and genera), the heatmap using Spearman correlation analysis was plotted using the online platform.^1^ With reference to Stegen et al. (2012), various ecological processes were divided based on phylogenetic and taxonomic diversity to analysis gut bacterial community assembly, which was conducted through the iCAMP package in R 4.2.3. At last, niche width gut bacterial community was calculated using *spaa* and *eoffice* packages in R 4.2.3, and visualized with Prism.

## 3 Results

### 3.1 Effects of organic fertilizer on functional traits of *Entomobrya proxima*

After cultivation for 120 days, the values of functional traits were all higher at the low addition concentration than in the control, albeit non-significant (*p* > 0.05). Higher body length, antenna length, middle leg length, and hind leg length were observed at the intermediate addition concentration compared to the control, with the significant difference found only in antenna length, while body width and foreleg length were lessened. In comparison with the control, high addition concentration increased body length and antenna length, yet diminished values of other parameters. Furthermore, for the three treatments added with organic fertilizer, treatment O1 promoted all functional traits except for antenna length, with significantly higher body width than treatment O2 and O3 (*p* < 0.05) and remarkably higher hindleg length than treatment O3 (*p* < 0.05). The order of antenna lengths was O2 > O1 > O3, but no significant difference was found among treatments. Excluding body length and body width, the remaining four parameters were lowest in treatment O3 (Table 1).

**Table 1:** Changes of functional traits of *E. proxima* under different treatments.

Functional trait length (mm)	Treatment			
	CK	O1	O2	O3
Body length	1.35 ± 0.03a	1.41 ± 0.02a	1.35 ± 0.03a	1.34 ± 0.03a
Body width	0.39 ± 0.02ab	0.42 ± 0.01a	0.36 ± 0.01b	0.36 ± 0.02b
Antenna length	0.53 ± 0.02b	0.58 ± 0.02ab	0.60 ± 0.02a	0.55 ± 0.03ab
Fore leg length	0.36 ± 0.02a	0.39 ± 0.01a	0.35 ± 0.01a	0.35 ± 0.02a
Middle leg length	0.45 ± 0.02a	0.46 ± 0.01a	0.46 ± 0.02a	0.42 ± 0.02a
Hind leg length	0.62 ± 0.03ab	0.68 ± 0.02a	0.63 ± 0.02ab	0.57 ± 0.03b

Data were presented as mean ± standard error (SE), and the different lowercase letters indicate the statistical significance (*p* < 0.05). Functional traits (*n* = 20).

### 3.2 Effects of organic fertilizer on the abundance, diversity and structure of gut bacterial community of *Entomobrya proxima*

Significant decline in gut bacterial abundance was observed in all treatments with organic fertilizer addition compared with the control (*p* < 0.05), and the absolute number decreased with increasing concentrations. Furthermore, gut bacterial community diversity, as indicated by both Chao1 and Shannon indices, was also lowered owing to the addition of organic fertilizer with greater decrease magnitude at low concentration than at both intermediate and high concentrations, and the Shannon index was significantly lower in treatment O1 than in the control (*p* < 0.05) (Table 2).

**Table 2:** Changes of gut bacterial community abundance, *α*-diversity of *E. proxima* under different treatments.

Abundance and *α-*diversity	Treatment			
	CK	O1	O2	O3
Abundance (10^6/ind.)	4.88 ± 0.27a	3.31 ± 0.26b	3.16 ± 0.15b	2.84 ± 0.46b
Chao1 index	380.38 ± 9.67a	300.20 ± 19.06a	376.89 ± 31.59a	345.76 ± 36.05a
Shannon index	3.93 ± 0.17a	2.99 ± 0.18b	3.55 ± 0.20ab	3.35 ± 0.24ab

Data were presented as mean ± standard error (SE), and the different lowercase letters indicate the statistical significance (*p* < 0.05). Abundance (*n* = 9), Chao 1 index and Shannon index (*n* = 3).

**Figure 2:**
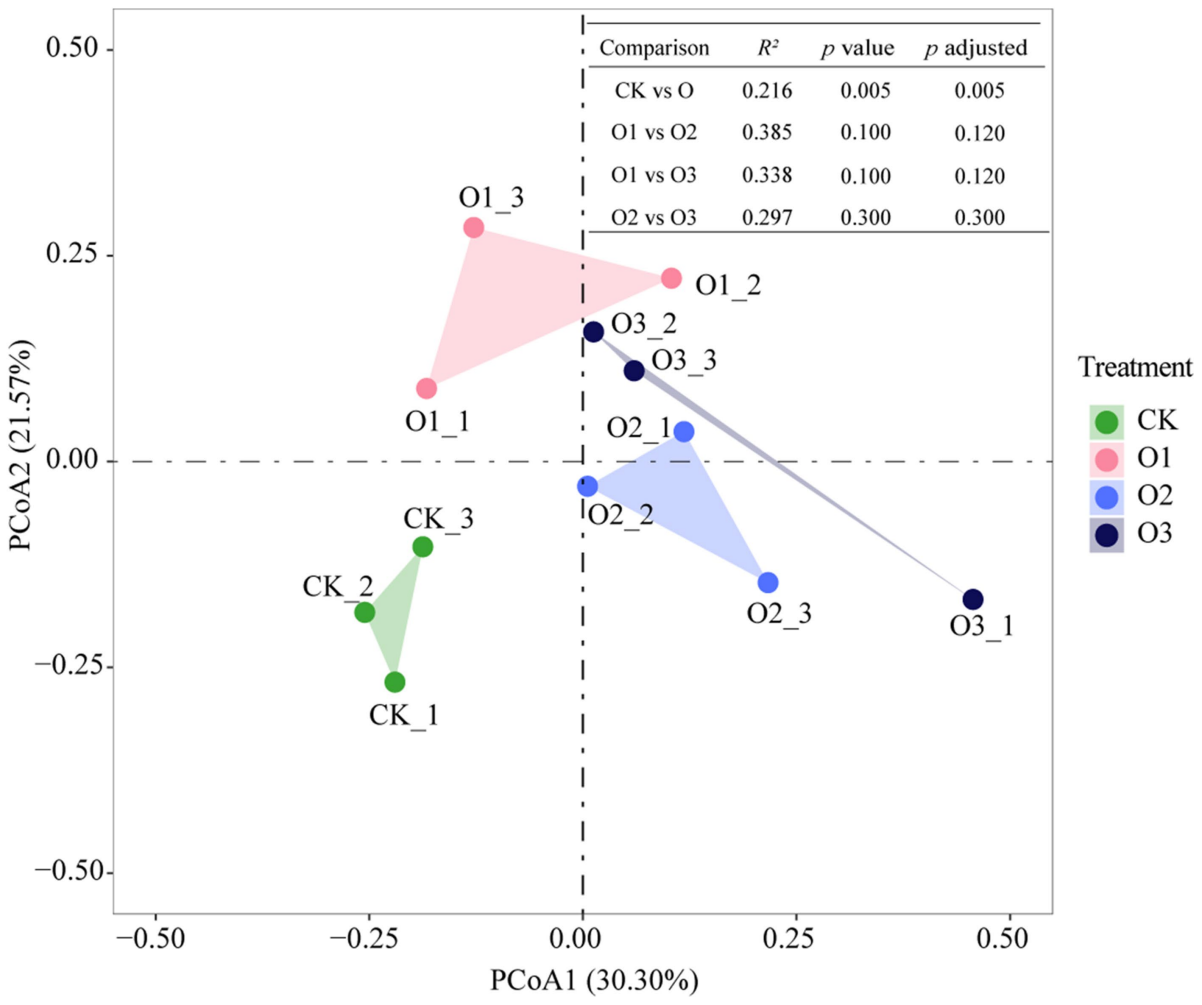
Changes of gut bacterial community structure of *E. proxima* under different treatments.

Joint analysis using Principal Coordinate Analysis (PCoA) and PERMANOVA tests revealed that gut bacterial community structure changed significantly in treatments with versus without organic fertilizer (*p* = 0.005), however, organic fertilizer concentration did not lead to significant variations in gut bacterial community structure, albeit obvious separation of these treatments from each other (Figure 2).

### 3.3 Effects of organic fertilizer on gut bacterial community composition of *Entomobrya proxima*

**Figure 3:**
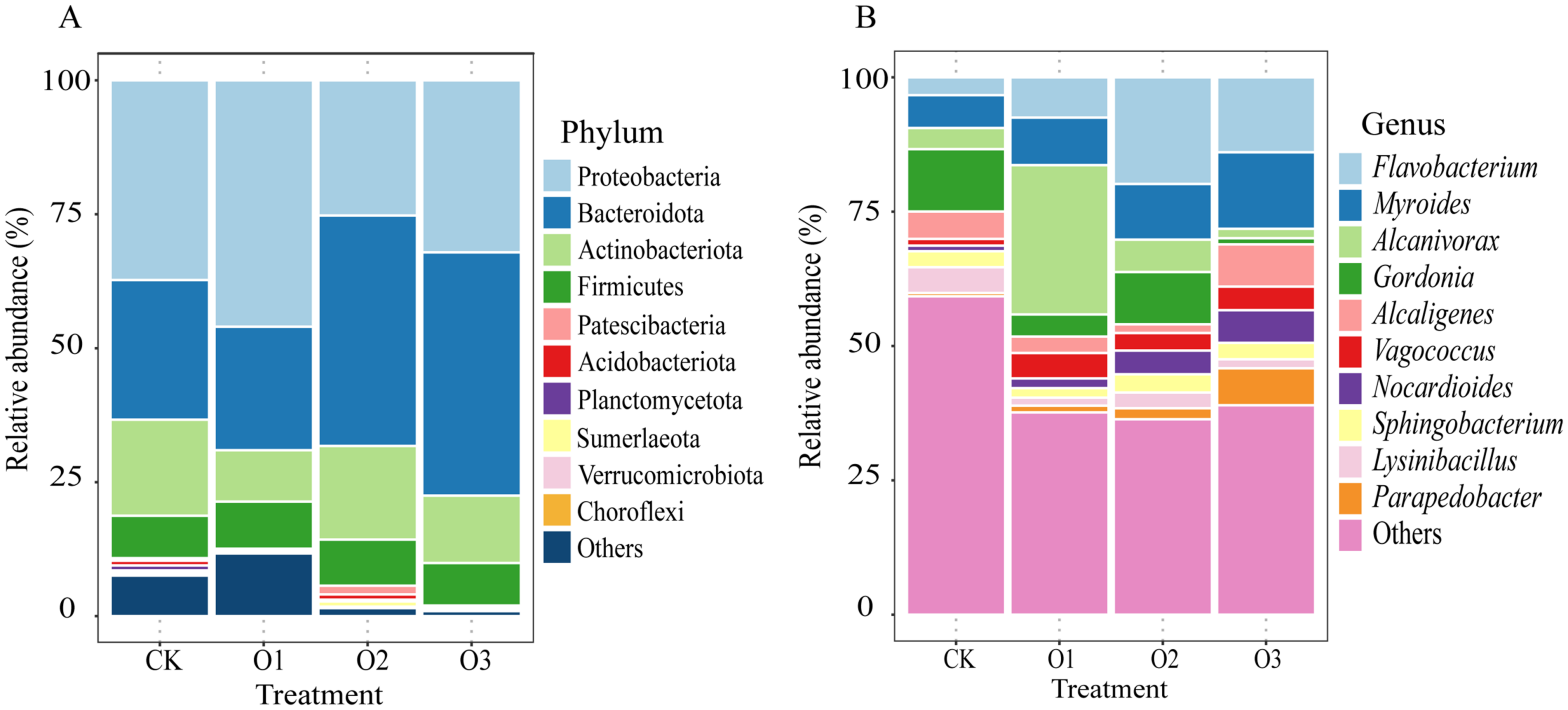
Changes of gut dominant bacterial community composition of *E. proxima* under different treatments, **(A)** phylum, **(B)** genus.

The top 10 phyla regarding relative abundance within the gut bacterial community of *E. proxima* are comprised of Proteobacteria (25.22–45.99%), Bacteroidota (23.05–45.41%), Actinobacteriota (9.59–17.93%), Firmicutes (7.93–8.80%), Patescibacteria (0.20%), Planctomycetota (0.16–0.93%), Acidobacteriota (0.06–1.00%), Sumerlaeota (0.06–0.98%), Verrucomicrobiota (0.08–0.41%), and Chloroflexi (0.08–0.37%) (Figure 3A). Significant proportion changes caused by the addition of organic fertilizer were noted for six phyla (one-way ANOVA, *p* < 0.05). In comparison with treatment CK, the relative abundance of Proteobacteria was increased by treatment O1 but was decreased by treatments O2 and O3, and there were significant differences only between treatments O1 and O2 (*p* < 0 0.05). A rise in the relative abundance of Bacteroidota was found in treatments with intermediate and high organic fertilizer concentrations compared to the control, especially for treatment O3 which exhibited a significant increase (*p* < 0 0.05). Conversely, treatment O1 lowered the proportion of Bacteroidota, and showed significant divergences compared with treatments O2 and O3 (*p* < 0 0.05). For Patescibacteria, a decrease in proportion was seen in treatments O1 and O3, and the high organic fertilizer concentration resulted in a great decrease magnitude (*p* < 0 0.05), while an opposite trend was observed in treatment O2, which substantially elevated the ratio compared with the other treatments added with organic fertilizer (*p* < 0 0.05). The relative abundance of Acidobacteriota in treatments O1 and O3 was significantly lower than that in treatment CK, which was true for that in treatment O2. Despite treatment O2 led to the rise of ratio of Acidobacteriota as compared to treatment CK, there was no significant change. The highest percentage of Sumerlaeota occurred at the intermediate concentration with significant differences, and the ratio in treatment CK was higher and lower than treatments O1 and O3, respectively. Moreover, a decreasing trend of Verrucomicrobiota was found as the concentration increased, with significant discrepancy at high input concentration compared with the control (*p* < 0.05).

The top 10 genera in terms of relative abundance within the gut bacterial community are as follows: *Flavobacterium* (3.31–19.82%), *Myroides* (6.11–14.24%), *Alcanivorax* (1.76–27.79%), *Gordonia* (1.13–11.63%), *Alcaligenes* (1.63–7.88%), *Vagococcus* (1.26–4.75%), *Nocardioides* (1.02–6.10%), *Sphingobacterium* (1.78–3.41%), *Lysinibacillus* (1.48–4.80%), and *Parapedobacter* (0.62–6.87%) (Figure 3B). Significant changes in relative abundances were observed for three genera because of organic fertilizer addition (one-way ANOVA, *p* < 0.05). The proportion of *Alcanivorax* was significantly increased at the low addition concentration compared to the control (*p* < 0.05), and also significantly higher than at intermediate and high concentrations (*p* < 0.05). The relative abundances of *Gordonia* and *Lysinibacillus* were both depressed by the treatments added with organic fertilizer, with significant decrease in treatment O3 (*p* < 0.05). Further, the percentage of *Gordonia* was highest in treatment O2, followed by treatments O1 and O3, while a significantly lower proportion of *Lysinibacillus* was observed in treatments O1 and O3 compared to treatment O2 (*p* < 0.05).

### 3.4 Effects of organic fertilizer on the assembly and niche breadth of intestinal bacterial community of *Entomobrya proxima*

**Figure 4:**
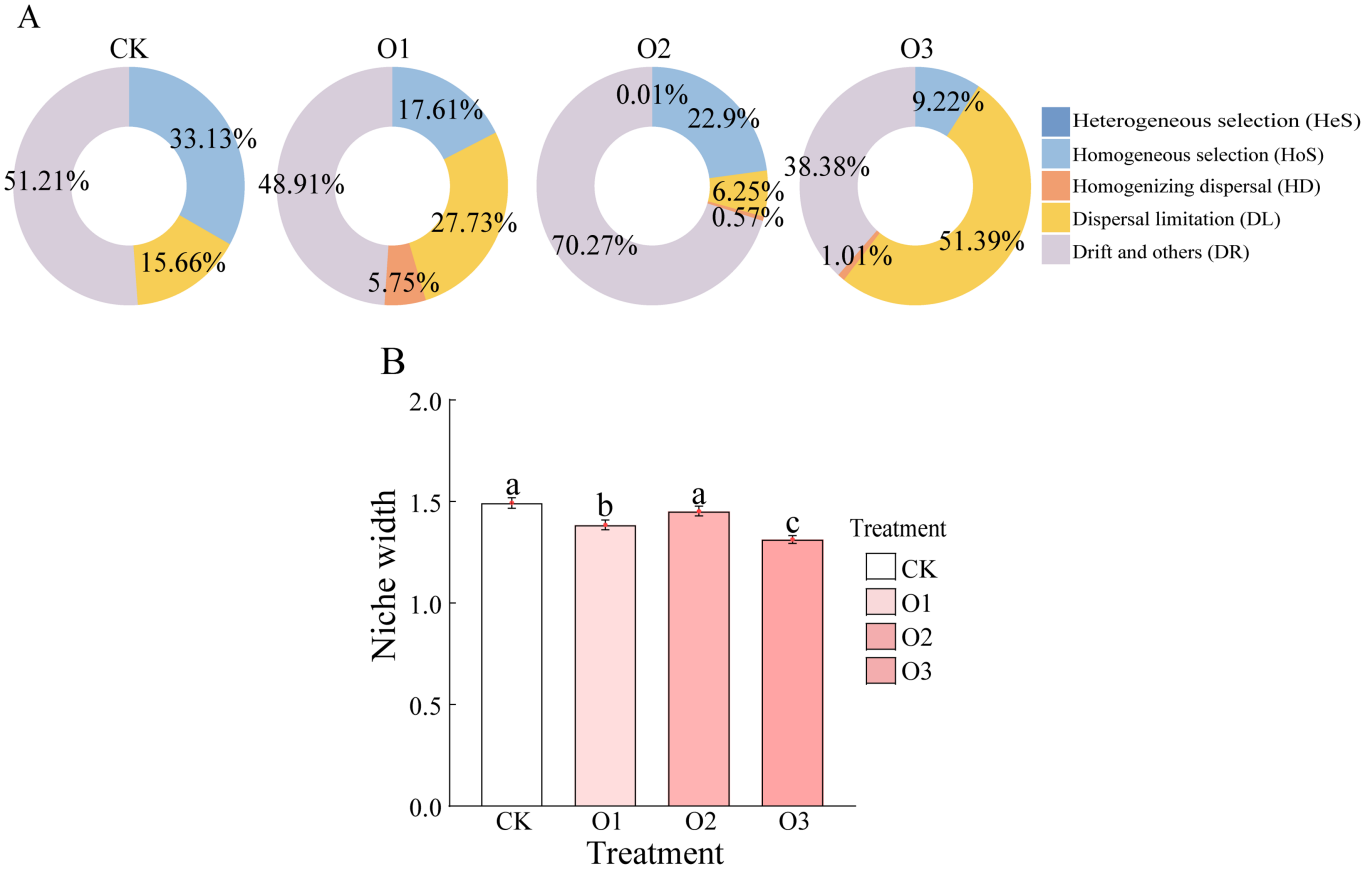
Changes of gut bacterial community assembly of *E. proxima* under different treatments, **(A)** infer community assembly mechanisms by phylogenetic-bin-based null model analysis (iCAMP), **(B)** niche width.

The iCAMP (Integrated Community Assembly Modeling Package) analysis indicated that drift and others (DR), dispersal limitation (DL) and homogeneous selection (HoS) co-drove the gut bacterial community assembly of all treatments (Figure 4A). However, the homogenizing dispersal (HD) was only found in treatments under organic fertilizer, and the contribution of HoS in organic fertilizer treatments was lower than that in treatment CK. Besides treatment O2, the relative importance of DL and DR under treatments O1 and O3 was higher and lower than treatment CK, respectively. With respect to niche width, the three treatments containing organic fertilizer all exhibited lower values compared to the control, with significant variations observed at low and high concentrations (*p* < 0.05). Nevertheless, the values of niche width in three organic fertilizer treatments were in the order: O2 > O1 > O3, with significant differences between treatments (*p* < 0.05) (Figure 4B).

### 3.5 Correlation analysis of functional traits and intestinal bacterial community

**Figure 5:**
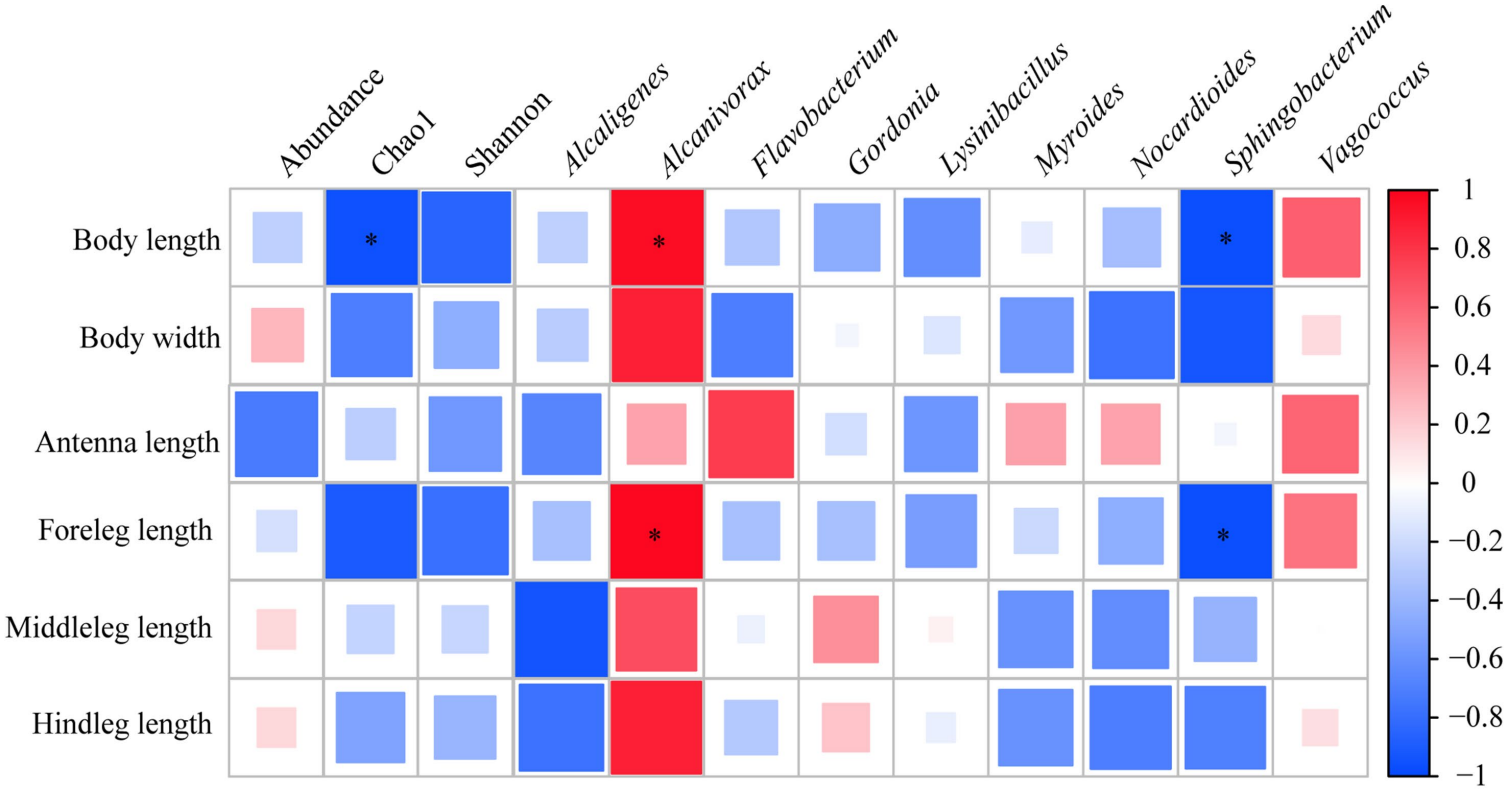
Spearman correlation analysis of functional traits and abundance, *α*-diversity indexes as well as relative abundances of top 10 genera of gut bacterial community of *E. proxima*. Red and blue indicate positive and negative correlation, respectively. Asterisk “*” represents significant correlation relationship (*p* < 0.05).

Correlation analysis based on Spearman rank correlation coefficient revealed that the Chao1 index of gut bacterial community exhibited a significant negative correlation with body length (*p* < 0.05). Furthermore, there was a significant positive correlation between relative abundance of *Alcanivorax* and body length and foreleg length (*p* < 0.05), however, the significant negative relationship was observed between *Sphingobacterium* and body length and foreleg length in sharp contrast (*p* < 0.05) (Figure 5).

## 4 Discussion

Organic fertilizer addition can influence functional traits of *E. proxima*. Compared to the control, the low addition concentration imposed a positive effect on all functional traits. Such enhancement may be attributed to more diverse food resources provided by organic fertilizer, consistent with the notion that organic fertilizer can facilitate the colonization of topsoil collembolans with greater exercises ability through enhanced food availability in topsoil (Yu et al., 2022). However, observed promotion did not consistently increase with increasing organic fertilizer concentrations, suppression as opposed occurred. Apart from antenna length, the functional trait values at the intermediate addition concentration were lowed to varying degrees compared to those at low addition concentration, yet the values of movement-related functional traits remained higher compared to the control, suggesting that traits with perception and exercise functions benefited from intermediate addition concentration. Considering that previous studies have shown that collembolans are sensitive to ammonium, and that the relative abundance of their gut bacterial community changes following the addition of fertilizers (Ding et al., 2019b), it is speculated that the introduction of high concentrations of organic fertilizer may lead to alterations in the relative abundance of the gut bacterial community in collembolans. Furthermore, there appears to be a potential correlation between the gut bacterial community and the functional traits of collembolans, which could result in changes across most functional traits, with the exception of antenna length. Nonetheless, still higher antenna length was the reflection of active foraging for *E. proxima* under this circumstance.

The addition of organic fertilizer not only altered the traits of *E. proxima*, but also resulted in reductions in both abundance and diversity of gut bacterial community. It is commonly believed that microbe rich soil provides substantial food resources available to collembolans, and organic input can enrich gut microbiota of collembolans via induction of changes in indigenous microbiota, i.e., indirect contribution to intestinal microbial community through the bottom-up cascade effect. However, it is important to note that in soil environment, biotic and abiotic properties, as well as their interrelation are highly dynamic, too much synergistic factors may lead to strong complexity. Hence, to elucidate the direct effect mechanism of organic fertilizer, a variety of edaphic influencing factors were not included and food resource types were minimized at the start of experiment based on the methodology internationally adopted. Consequently, declined abundance and diversity of gut microbiota was the direct and real reflection of organic fertilizer impacts on the collembolan selected. In addition, organic fertilizer caused significant changes in bacterial community structure, which was obviously separated under different concentrations of organic fertilizer, but there was no significant difference. It was speculated that the gut bacterial community responded to the same fertilizer with different concentrations in the same way.

On the other hand, disturbances to gut bacterial community of *E. proxima* by ingestion of organic fertilizer was also reflected in the composition alterations. At the phylum level, low addition concentration increased the relative abundance of Proteobacteria. Microorganisms within this phylum have been reported to be able to stimulate the production of flagellin and lipopolysaccharide that are capable of inducing inflammatory responses (Guo et al., 2022), suggesting that such microbe regulatory stress responses may occur due to the increased proportion of Proteobacteria, which may be detrimental to intestinal homeostasis. In the context of organic addition at intermediate and high concentrations, there was an increase in the relative abundance of Bacteroidota, which are reported to have a potential role in degrading carbohydrates and proteins and are speculated to help host enhance digestion and nutrient absorption efficiency (Pitta et al., 2016). Additionally, Patescibacteria with a strong adaptability to adverse environments (Tian et al., 2020) were observed to receive a notable boost from organic fertilizer, which implied that the formed habitat conditions were more conductive to the propagation of gut microbiota that are tolerant to unfavorable environments, resulting in the creation and maintenance of intestinal homeostasis. Additionally, multiple studies have reported that Acidobacteriota are vulnerable to acidity and temperature (Zhao et al., 2024), hence, significant decreases of their proportion could be the result of shifts in acid–base microenvironment within the intestinal tract under the circumstances of low and high addition concentrations of organic fertilizer.

Further analysis at the genus level revealed that the addition of organic fertilizer significantly increased the relative abundance of *Alcanivorax*, while remarkably decreased that of *Gordonia*. Both genera have been demonstrated to have the ability of alkane degradation (McGenity and Laissue, 2023; Sowani et al., 2018). The elevated ratio of *Alcanivorax* was found to potentially promote the development of functional traits. Given that both *Alcanivorax* and *Gordonia* possess alkane-degrading capabilities, their abundance changes may differ due to competition or environmental adaptation. It was speculated that the relative abundance of *Alcanivorax* increased in response to the alkanes produced by gut bacteria following the addition of organic fertilizer, which may help maintain intestinal homeostasis and thereby benefit the elongation of functional traits. Although it was observed that the relative abundance of *Sphingobacterium* was negatively correlated with all functional traits, especially significantly with body length and forefoot length, organic fertilizer input did not cause a dramatic shift in its proportion, indicating significant functional trait alterations caused by slight changes of this genus.

The way of *E. proxima* responded to organic fertilizer addition was also revealed by variations in its gut bacterial community assembly and niche width. Organic fertilizer application decreased the contribution of Hos and increased the drive of HD. Considering that HoS means that the microbiota tends to be occupied by taxa with similar ecological niches, and HD is conducive to homogenization between communities (Zhou and Ning, 2017). It is inferred that the application of organic fertilizer caused slow turnover and was conductive to stability of gut bacteria of collembolans. This finding could be confirmed by isotope labeling in subsequent study. Besides, the drive of DL was enhanced and the niche width of gut bacterial community was decreased significantly under treatments O1 and O3. The drive of DL can lead to increased microbial community heterogeneity and niche narrowing suggests increased available resources owing to diversified food sources, resulting in a more specialized gut bacterial community with specific environmental adaptability (Xu et al., 2022). It was speculated that low and high organic fertilizer addition made gut bacterial communities more diverse and complex, and gradually showed specific environmental adaptability. Besides, DR is recognized as the significant contributor to biodiversity loss (Gilbert and Levine, 2017), and this driver effect is much higher under treatment O2 than other treatments. Considering that the niche width was not significantly different from that of treatment CK, it was speculated that the microbial diversity was reduced and no specific environmental adaptability appeared under treatment O2. Narrower niche width was associated with elevated species richness, suggesting that more resources were available in the environment (Granot and Belmaker, 2020). The relative abundance of *Lysinibacillus* was consistent with changes in niche width, being lower under treatments O1 and O3 compared to treatment O2. This decrease in *Lysinibacillus* abundance may lead to an increase in available resources, and potentially enhancing species richness (Zeng et al., 2023). However, the specific mechanisms underlying these relationships require further investigation.

In this experiment, soilless culture was conducted in an indoor environment. Although the interference of various substances in the soil environment was excluded, the interaction of abundant soil animals and microflora in the actual soil environment and the changes of gut microflora in the decomposition process of organic fertilizer on collembolans could not be investigated. And for the actual field application of organic fertilizer and the stability of functional traits of soil animals, the test period of 120 days is too short. Furthermore, future studies should consider incorporating soil and fertilizer perturbations (including organic, inorganic, and compound fertilizers), extending the experimental duration, and employing multi-omics technologies to investigate the interactive pathways between host gut microbiota and functional traits. This approach would comprehensively elucidate the impacts of fertilizers on soil fauna and their gut microbiota, thereby offering valuable insights for maintaining soil health and promoting sustainable agricultural development.

## 5 Conclusion

The effects of organic fertilizer on soil fauna were examined based on a 120-day cultivation experiment involving the soil collembolan (*E. proxima*). The influence of organic fertilizer on functional traits varied across different concentrations, demonstrating a tendency for low promotion and high inhibition. Consequently, *E. proxima* adopted strategies such as reducing population density and increasing gut microbiota diversity to adapt to changes in food resources. Furthermore, the structure of the gut bacterial community was significantly altered; however, similar structural patterns were observed across different concentrations. The application of organic fertilizer induced variations in gut bacterial community composition at both phylum and genus levels. Notably, functional traits exhibited a positive correlation with the relative abundance of *Alcanivorax* but a negative correlation with that of *Sphingobacterium*, indicating their potential as biomarkers. The concentration of organic fertilizer emerged as a key determinant influencing gut bacterial community assembly. Concurrently, there was a narrowing of niche width, which may result in an increased dependence on specific food sources. In summary, *E. proxima* responded to the application of organic fertilizer by altering its functional traits and gut bacterial community; however, the effects varied according to input ratios. Further research is warranted to fully elucidate the underlying mechanisms involved.

## Data Availability

The sequencing data were submitted to NCBI under Bio Project PRJNA1196609.

## Footnotes

^1^ cloudtutu.com.cn
